# Acute Guillain-Barré polyradiculoneuritis indicative of COVID-19 infection: a case report

**DOI:** 10.11604/pamj.supp.2020.35.150.25745

**Published:** 2020-08-27

**Authors:** Hugues Ghislain Atakla, Mahugnon Maurel Ulrich Dénis Noudohounsi, Hélène Sacca, Nana Rahamatou Aminou Tassiou, Wilfried Cadnel Noudohounsi, Dismand Stephan Houinato

**Affiliations:** 1 Neurology Department, Ignace Deen University Hospital Center, Conakry, Guinea,; 2 Public Health Physician, Research Project Manager, Brazzaville, Congo,; 3 Laboratory of Noncommunicable and Neurologic Diseases Epidemiology, Faculty of Health Science, University of Abomey-Calavi, Cotonou, Benin,; 4 Neurology Department, Hubert Koutoukou Maga University Hospital Center, Cotonou, Benin,; 5 Neurology Department, Centre Hospitalier sud francilien, France

**Keywords:** Guillain Barré polyradiculoneuritis, COVID-19, case report, Guinea

## Abstract

The new coronavirus 2019 epidemic declared in China on December 31, 2019 soon spread to the rest of the world, becoming the subject of an unprecedented health pandemic according to the World Health Organization's declaration of March 11, 2020. It is a disease that has the potential to cause multiple systemic infections. We report here the case of an acute polyradiculoneuritis of the Guillain-Barré type (GBS) indicative of a COVID-19 infection. This is a 41 year old patient seen for ascending, symmetrical and bilateral, progressive and acute tetraparesis with in a context of influenza syndrome and digestive infections treated 2 weeks earlier. During a COVID-19 infection, certain inflammatory cells stimulated by the virus produce inflammatory cytokines creating immune-mediated processes. The same mechanism is observed in GBS being also an immune-mediated disorder. The management of this disease in COVID-19 positive patients does not differ from that of patients who do not carry the virus. The risk of respiratory distress in COVID-19 positive patients becomes twice as great in patients with GBS who test positive for COVID-19 at the same time. Monitoring for hemodynamic disorders and respiratory distress in a neuro-intensive care unit may be fruitful.

## Introduction

The recent highly contagious coronavirus pandemic remains a daily concern for clinicians and researchers around the world. The latter condition, related to the novel coronavirus (COVID-19), was detected in Wuhan (Hubei), a province in China, on December 31, 2019 [1]. After an incubation period of 5 days, the most frequent symptoms of the disease are: fever, cough, myalgia, dyspnea, headache and diarrhea [2]. Gastrointestinal and cardiovascular complications related to COVID-19 are also frequently reported in the literature [3,4]. The same is true for the central and peripheral nervous system with complications such as ischemic or hemorrhagic stroke [5,6]. Silas Webb *et al*. and Donatella Ottaviani *et al*. have each reported one case of Guillain-Barré Syndrome (GBS) following COVID-19 infection [7,8]. Series of cases of GBS linked to COVID-19 have been reported in China, Italy, Iran and the United States [9-11], but this is the first case described in the Republic of Guinea. Guillain-Barré syndrome is an acute polyradiculoneuritis caused by various infections [12]. Typically, the clinical manifestations of GBS consist of a flaccid, progressive, ascending and symmetrical paralysis of all four limbs accompanied by sensory and/or vegetative disturbance [13]. One of the serious complications of the disease is respiratory distress due to paralysis of the respiratory muscles by involvement of the bulbar cranial nerves. The risk of respiratory failure and the rapid progression of GBS represents a health emergency that requires early diagnosis and follow-up in a neuro-intensive care unit. [Here we describe the case of a 41-year-old patient with a clinical picture characteristic of GBS whose history suggests that the RT-PCR of the nasopharyngeal swab was positive while that of the cerebrospinal fluid (CSF) was negative].

In agreement with the National Health Safety Agency (ANSS) prospective data on the patient were obtained with his own informed consent. In this work, we report a case of infection with a new coronavirus insidiously revealed in a patient suffering from acute Guillain-Barré polyradiculoneuritis.

## Patients and observation

A 41 year old man was admitted to the emergency room with a rapidly progressive, symmetrical and ascending motor deficit of all 4 limbs, evolving in a picture of respiratory distress with a drooping head that had been evolving for 4 hours on admission. The anamnesis reveals that the clinical manifestations would have started with paresthesias of the type of tingling of the lower limbs, rapidly giving way to a progressive weakness of the lower limbs starting at the distal extremities 96 hours before his admission. The muscle weakness evolved symmetrically and bilaterally from the distal muscles to the proximal muscles and the patient thus became tetra paretic 24 hours before admission. The hetero anamnesis collected from his family reported that the patient had presented an influenza syndrome accompanied by a digestive disorder related to salmonellosis that had retroceded to β-lactamine (amoxicillin) and fluoroquinolone (ciprofloxacin) the previous 2 weeks. He reported as another complaint a loss of smell and taste one week before the onset of sensory and motor manifestations. In addition, there was no known notion of contagion. Clinical examination revealed a fever of 38.5°C, hypersudation, tachycardia at 113 beats/minute, polypnoea at 28 cycles/minute, blood pressure at 111/70 mmhg and oxygen saturation at 89%. The patient was conscious and had no dyspnea on admission. Examination of segmental muscle strength to show 4 limb weakness with a Medical research council (MRC) scale of 2/5 proximal and 1/5 distal of the lower extremities and 3/5 both proximal and distal of the upper extremities. Osteotendinous reflexes were decreased in all 4 limbs. There was facial diplomegia and velo-pharyngeal paralysis with difficulty swallowing. The sensitivity study revealed a deep sensitivity disorder of the vibratory type with preservation of superficial sensitivity. There was no spinal injury level, cognitive function was preserved, no evidence of meningeal irritation, no urinary or fecal incontinence.

**Figure 1:**
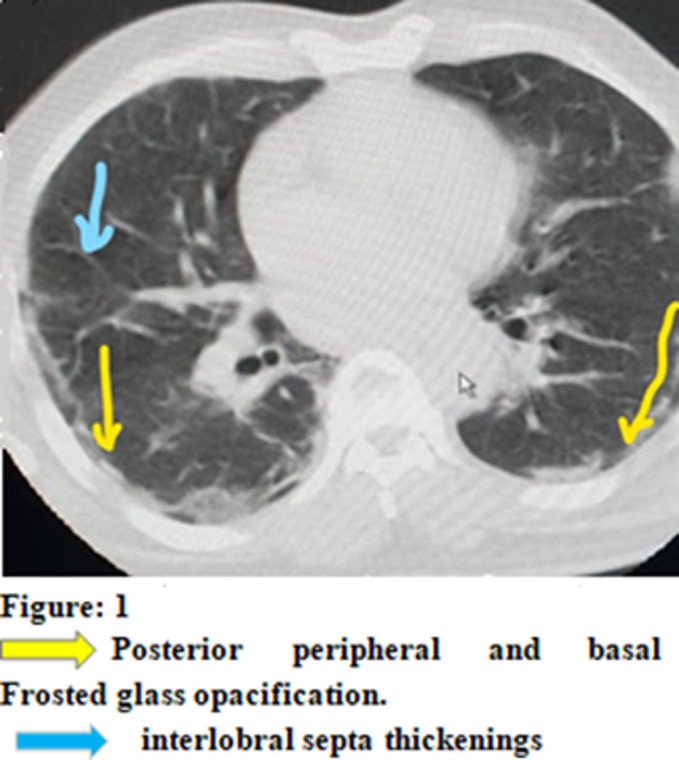
posterior peripheral and basal frosted glass opacification. Interlobral septa thickenings

Paraclinically, the RT-PCR test result was positive in the nasopharyngeal swab although the LCS was negative. The patient received a chest CT scan that revealed images suggestive of COVID-19 (Figure 1). The ECG showed complete tachyarrhythmia with atrial fibrillation. Other laboratory findings were hemoglobin 12.7g/dl, white blood cells 6280/mm^3^with deep neutropenia at 500 mm^3^. The sedimentation rate (SV) was: 36 mm at the 1^st^hour and the C-reactive protein was increased to 86 mg/l. Other tests for endemic infections in our media were performed but all were negative. CSF analysis showed albumino-cytological dissociation: with hyperproteinorrhage at 64 mg/dl and normal glycorachia and lymphocytes/mm^3^. No viral agents were found on Gram stain and viral PCR including SARS-Cov-2 RNA was negative. MRI of the cervical spine showed no pathological findings. Motor conduction evaluation in ENMG (electroneuromyogram) performed 72 hours after admission showed prolonged distal motor latencies. Stimulation of the tibial nerves induced normal F-wave latencies with pathological intermediate latency responses of complex A-wave on both sides. Sensory conduction studies of the median, ulnar and sural nerves and motor nerve conduction studies of the median, ulnar and tibial nerves were normal. Since electromyography (EMG) showed no evidence of axonal injury (denervation), we suggested the diagnosis of acute inflammatory demyelinating polyradiculoneuritis secondary to an SARS-Cov-2 infection.

After isolation, the patient was put under monitoring, nasal oxygen therapy, mechanical ventilation, nasogastric and urinary catheterization. He received 0.4g/kg/day of immunoglobulin for 5 days before being relayed with Azythromycin 500mg and nursing was strictly observed. However, he did not benefit from the standard treatment against COVID-19 (Chloroquine) in Guinea because of the rhythm disorder observed on the ECG. Respiratory function became normal in less than 48 hours, with regression of the swallowing disorder after 8 days of hospitalization. No significant improvement in sensory-motor and vegetative disorders was observed during the first week. However, at 16 days of hospitalization there was a marked improvement in the patient's neurological state, with segmental muscle strength rated 3/5 proximal and distal to the lower extremities and 2/5 proximal and 3/5 distal to the lower extremities according to the MRC scale. There was also a regression of the pallesthesia disorder which was present only in the upper limbs and persistence of urinary incontinence. At 28 days of hospitalization, the patient was asymptomatic at COVID-19 and the muscle strength rating was 4/5 at all 4 limbs. Following a negative RT-PCR on 2 occasions, the patient was transferred to a physiotherapy unit for active rehabilitation.

The patient reports satisfaction with early diagnosis, follow-up and regular nursing. However, he deplores the persistence of his urinary incontinence and the weakness of his limbs after 4 weeks of hospitalization.

## Discussion

In this study, we reported one case of GBS indicative of COVID-19 infection. According to the literature, the first case of GBS associated with CoV-2-SARS infection was probably reported by Zhao *et al*. who reported a positive COVID-19 test in a 61-year-old woman with GBS [9]. The clinical manifestations of GBS found in this presentation are well known and clearly described in the literature [14,15]. Several authors have already reported the onset of neurological symptoms such as anosmia and agueusia 5 to 10 days before the onset of neurological complications of COVID-19 [16,17]. In the clinical data of five patients with GBS who tested positive for COVID-19 analyzed by Toscano *et al*., three of them had already suffered from anosmia or agueusia [17]. Although the etiopathogeny of GBS has been poorly understood, the disease is usually preceded by a respiratory or gastrointestinal infection. Various viral agents have been associated with GBS (cytomegalovirus, Campylobacter jejuni, Epstein-Barr virus, and Zika virus. In this work, we have reported an association between GBS and the novel coronavirus. To these groups of agents already identified, we can reasonably add the new coronavirus. The interval of 5 to 10 days between the onset of respiratory symptoms of the disease and the first manifestations of GBS in our case is similar to the cases reported by Virani *et al*. and Toscano *et al*. [17,18]. Given the context, we hypothesize that there is a causal relationship between GBS and SARS-Cov-2 infection. However, there is no evidence of direct nerve root invasion by the virus, as the RT-PCR test performed on the LCS swab was negative. The nerve conduction study allowed us to rule out axon-demyelinating sensory-motor polyneuropathy, among other things.

The patient received the same treatment as patients with GBS apart from COVID-19 infection. Chloroquine commonly administered in COVID-19 positive patients in Guinea was not used in this patient because of the heart rhythm disorder observed on ECG and the high risk of heart rhythm disorder associated with the use of this drug in the healthy patient. The remainder of the management was purely symptomatic. In this report the functional recovery with persistent residual weakness observed after 4 weeks of treatment and follow-up is comparable to the observation of Mohammad Amin Farzi [15]. It is thought that the damage induced by the inflammatory cascade on the nerve roots takes time to resolve even under conditions of early management. Furthermore, since GBS symptoms have been reported as telltale signs of COVID-19, it becomes crucial that clinical physicians keep this association in mind in order to quickly initiate treatment and clinical monitoring. Like Mao Ling *et al*. [6] in China, the authors agree that special attention should be paid to the neurological complications of COVID-19, especially in those affected by GBS. Although the analysis of the CSF sample was negative to RT-PCR, we believe that GBS is a consequence of COVID-19 infection since the virus was detected in the nasopharyngeal swab. Other tests for endemic infections in our settings were performed but all were negative. An upcoming large-scale study of all GBS cases diagnosed during this pandemic will further elucidate the pathogenesis of this disease.

## Conclusion

In summary, we say that GBS also occurs in patients with COVID-19 who have had no previous respiratory symptoms. Analysis of RT-PCR negative CSF in a patient who is positive on nasopharyngeal swab should not delay management given the risk of respiratory distress and sudden death related to the disease. Early diagnosis and management in the resuscitation unit is necessary to optimize the patient's chances of survival.
